# Head to head comparison of [^99m^Tc]Tc(CO)_3_(NTA) and [^99m^Tc]Tc-MAG3 in patients with suspected obstruction

**DOI:** 10.1186/s13550-021-00782-x

**Published:** 2021-05-01

**Authors:** Andrew T. Taylor, Malgorzata Lipowska, Raghuveer K. Halkar

**Affiliations:** Department of Radiology and Imaging Sciences, Emory University, Atlanta, GA 30322 USA

**Keywords:** Diuretic renography, Renal radiopharmaceuticals, ^99m^Tc-tricarbonylnitrilotriacetic acid, ^99m^Tc(CO)_3_(NTA), NTA, ^99m^Tc-mercaptoacetyltriglycine, ^99m^Tc-MAG3, MAG3

## Abstract

**Purpose:**

^99m^Tc-tricarbonyl-nitrilotriacetic acid, [^99m^Tc]Tc(CO)_3_(NTA), is a new ^99m^Tc-renal radiopharmaceutical with a clearance equal to that of ^131^I-ortho-iodohippuran, [^131^I]I-OIH. Our purpose was to compare the performance of [^99m^Tc]Tc(CO)_3_(NTA) and [^99m^Tc]Tc-MAG3 in patients with suspected obstruction.

**Methods:**

[^99m^Tc]Tc(CO)_3_(NTA) was prepared with commercially available NTA ligand and CRS Isolink kit, and isolated by HPLC. Eighteen adult patients referred for diuretic renography received an intravenous injection of approximately 40 mg of furosemide 15 min prior to either [^99m^Tc]Tc(CO)_3_(NTA) or [^99m^Tc]Tc-MAG3 (mean activity of 47 ± 4.4 MBq). Data were acquired for 24 min followed by an anterior image of the liver and gall bladder and a measure of voided volume. Patients received a second furosemide injection equal to one third of the original dose followed fifteen minutes later by administration of the alternate tracer, mean activity of 320 ± 34 MBq. Clearances were measured using a camera-based technique.

**Results:**

The clearance of NTA was greater than that of MAG3, 331 ± 146 versus 271 ± 105 mL/min/1.73 m^2^, respectively, *p* < 0.0001. The kidney to background ratio for NTA was greater than that of MAG3 for both left and right kidneys, *p* < 0.001; the 20 min/maximum count ratio was significantly less, *p* < 0.0001. There was no significant difference in the voiding volumes following NTA and MAG3 administration, 598 ± 237 mL versus 498 ± 170 mL, respectively, *p* = 0.07. Gall bladder activity was not observed with NTA but was present in 6/17 MAG3 studies. Images and renogram curves were comparable except for two patients where the NTA study excluded obstruction but the MAG3 study suggested an indeterminate or obstructed kidney.

**Conclusions:**

Unlike MAG3, NTA is not eliminated via the hepatobiliary track. Moreover, NTA has a higher kidney to background ratio and more rapid clearance than MAG3. These advantages should allow more robust camera-based clearance measurements and may lead to better discrimination between obstructed and non-obstructed kidneys.

## Introduction

Adult and pediatric consensus groups consider ^99m^Tc-mercaptoacetyltriglycine, [^99m^Tc]Tc -MAG3 (MAG3) to be superior to ^99m^Tc-diethylenetriaminepentaacetic acid, [^99m^Tc]Tc-DTPA (DTPA), for diuresis renography [1–5]. MAG3 has a much higher extraction efficiency than DTPA; consequently, less MAG3 remains in circulation during the washout period and less is entering the kidney at the time washout is evaluated [6, 7].

^99m^Tc-tricarbonyl-nitrilotriacetic acid, [^99m^Tc]Tc(CO)_3_(NTA) (NTA), is a new tubular tracer with a clearance equal to that of ^131^I-orthoiodohippuran, [^131^I]I-OIH (OIH); in contrast, the clearance of MAG3 is only 50–60% that of OIH [6–9]. Moreover, a small percentage of the injected dose of MAG3 is eliminated via the hepatobiliary pathway and that percentage increases as renal function deteriorates [6–11]. Activity in the gall bladder has been mistaken for renal activity and gut activity arriving via the hepatobiliary pathway may be interpreted as a urine leak [12, 13]. NTA, in contrast, has shown no hepatobiliary elimination [8, 9].

Because NTA is cleared more rapidly than MAG3 and lacks hepatobiliary elimination, we hypothesized that NTA might be superior to MAG3 in patients with suspected obstruction for the same reasons that MAG3 is superior to DTPA. The objective of this investigation was a direct comparison of NTA and MAG3 in this clinically relevant patient population.

## Materials and methods

### ***Radiosynthesis of [***^***99m***^***Tc]Tc(CO)***_***3***_***(NTA)***

Nitrilotriacetic acid (NTA ligand) was purchased from Aldrich and labeled as previously described [14]. ^99m^Tc-pertechnetate ([^99m^Tc]TcO_4_^−^) in 0.9% saline was received from Triad Isotopes (Norcross, GA, USA). The “CRS Isolink kit” (Center for Radiopharmaceutical Science, Paul Scherrer Institute, Villigen, Switzerland) was used to prepare the [[^99m^Tc]Tc(CO)_3_(H_2_O)_3_]^+^ precursor according to the manufacturer’s insert. [^99m^Tc]Tc(CO)_3_(NTA) was separated from unlabeled ligand by high-performance liquid chromatography (HPLC) instrument (System Gold Nouveau, Beckman Coulter, Brea, CA) equipped with a model 170 radiometric detector and a model 166 ultraviolet light-visible light detector, 32 Karat chromatography software (Beckman Coulter), and an octyldecyl silane column (C18 RP Ultrasphere; 5-µm, 4.6 × 250 mm; Beckman Coulter). The solvent system was 0.05 M TEAP buffer pH 2.5 (solvent A) and ethanol (solvent B) and the flow rate was 1 mL/min; the radiochemical purity was > 99%. The gradient method was the same as reported previously [15].

Ethanol was partially removed by N_2_ gas, and the collected solution of [^99m^Tc]Tc(CO)_3_(NTA) was buffered in a physiological phosphate buffer (PBS) at pH 7.4. The HPLC-purified complex in PBS (pH 7.4) was passed through a sterile Millex-GS 22 µm filter (Millipore Co., St Louis, MO) (primed with 4 mL of saline) into a sterile, pyrogen-free empty vial. The final concentration was 74–148 MBq/mL (2–4 mCi/mL) and the final pH was 7.4. Test samples were sent for analysis and determined to be sterile and pyrogen free.

### Patient studies

An eIND was obtained from the FDA and all studies were performed with the approval of the Radiation Safety Committee for Human Use of Radiation (RSC1) and the Institutional Review Board. Signed consent was obtained from each participant. Adult patients referred by urologists for diuretic renography were asked to participate. Nineteen subjects entered the protocol but one withdrew after signing the consent due to lack of adequate venous access. Each of the remaining 18 subjects (mean age 51.3 ± 16.8 years, 11 females, 7 males) was monitored with measurements of blood pressure, heart rate, and temperature obtained before and after the study; they were under constant observation during the study and were further monitored via a follow-up phone call 24 h later. Each patient drank 200 mL of water on arrival; an intravenous line was connected to a one-liter bag of normal saline for fluid replacement during the diuretic portion of the study.

Both studies were performed on the same day using a protocol similar to that of a two-stage ACE inhibition study [16]; a low activity acquisition was followed by a high activity acquisition to minimize the effect of residual activity on calculations derived from the second acquisition. Our intention was to alternate the order of NTA and MAG3 administration but the HPLC preparation of [^99m^Tc]Tc(CO)_3_(NTA) rarely yielded a high enough concentration of activity to allow 300–370 MBq of [^99m^Tc]Tc(CO)_3_(NTA) to be administered as a tight bolus. To minimize the volume of injectate and maintain a tight bolus, NTA was administered first as the low activity acquisition in 16/18 subjects. The mean activity of the initial tracer administrated was 47 ± 4.4 MBq; the mean activity of second tracer was 320 ± 34 MBq, The majority of patients received an intravenous injection of 40 mg of furosemide 15 min prior to the administration of first tracer and 14 mg of furosemide prior to administration of the second tracer to maintain a comparable serum concentration of the diuretic [17]. Exceptions were patients 10 (60 mg and 30 mg), patient 16 (30 mg and 10 mg) and patient 17 (80 mg and 27 mg).

Imaging was performed for 24 min following tracer injection using a General Electric Infinia camera (Milwaukee, WI) with a 20% window centered over the 140 keV photopeak of ^99m^Tc. Data were acquired in a 128 × 128 matrix by using a three-phase dynamic acquisition and processed on a General Electric Xeleris computer using non-commercial, in house update of the QuantEM™ renal software. Kidney regions of interest were automatically assigned and could be modified by the operator; background was automatically assigned as a peri-renal region of interest 2 pixels wide and one pixel outside of the renal ROI [18].

The acquisition was followed by an anterior image of the liver and gall bladder, and a measurement of voided volume. Patients drank an additional 200 mL of water before receiving the second injection of furosemide. Fifteen minutes following furosemide, the alternate tracer was administered and the same acquisition protocol was utilized. Intravenous hydration was continued throughout both acquisitions. Renogram curves were generated using whole kidney regions of interest (ROIs). Clearances were measured using a camera-based technique and the kidney to background ratios were calculated from the same summed frames (1–2.5 min after injection) used to calculate relative uptake and the camera-based clearance [19].

Two subjects had only one kidney. Six of 18 subjects had to void prior to completing the first 24 min acquisition; all of these patients received NTA. Two patients had to void prior to completing the second acquisition, one following MAG3 and one following NTA, and two studies demonstrated marked NTA tracer infiltration at the injection site. Comparisons impacted by dose infiltration or incomplete 24 min acquisitions were not included in the data analysis.

The camera-based clearance method in this study utilized a regression equation developed for MAG3 that converts the percent injected dose in the kidney at 1–2.5 min post injection to a clearance expressed in mL/min/1.73 m^2^ [19]. To test the applicability of this regression equation for NTA, we used the MAG3 regression equation to calculate the camera-based clearance of NTA from two previous studies in which the NTA clearance was calculated determined using the single-injection, 2-compartment model of Sapirstein et al. [8, 9, 20]. These two studies contained 17 subjects, 9 volunteers with normal renal function and 8 with a diagnosis of chronic kidney disease [8, 9].

### Statistical analysis

All results were expressed as the mean ± SD. To determine the statistical significance of differences between the 2 groups, comparisons were made with the 2-tailed Student *t* test for paired data; *p* < 0.05 was considered statistically significant. The concordance correlation coefficient was used to compare the camera-based and plasma-based NTA clearances [21].

## Results

**Fig. 1:**
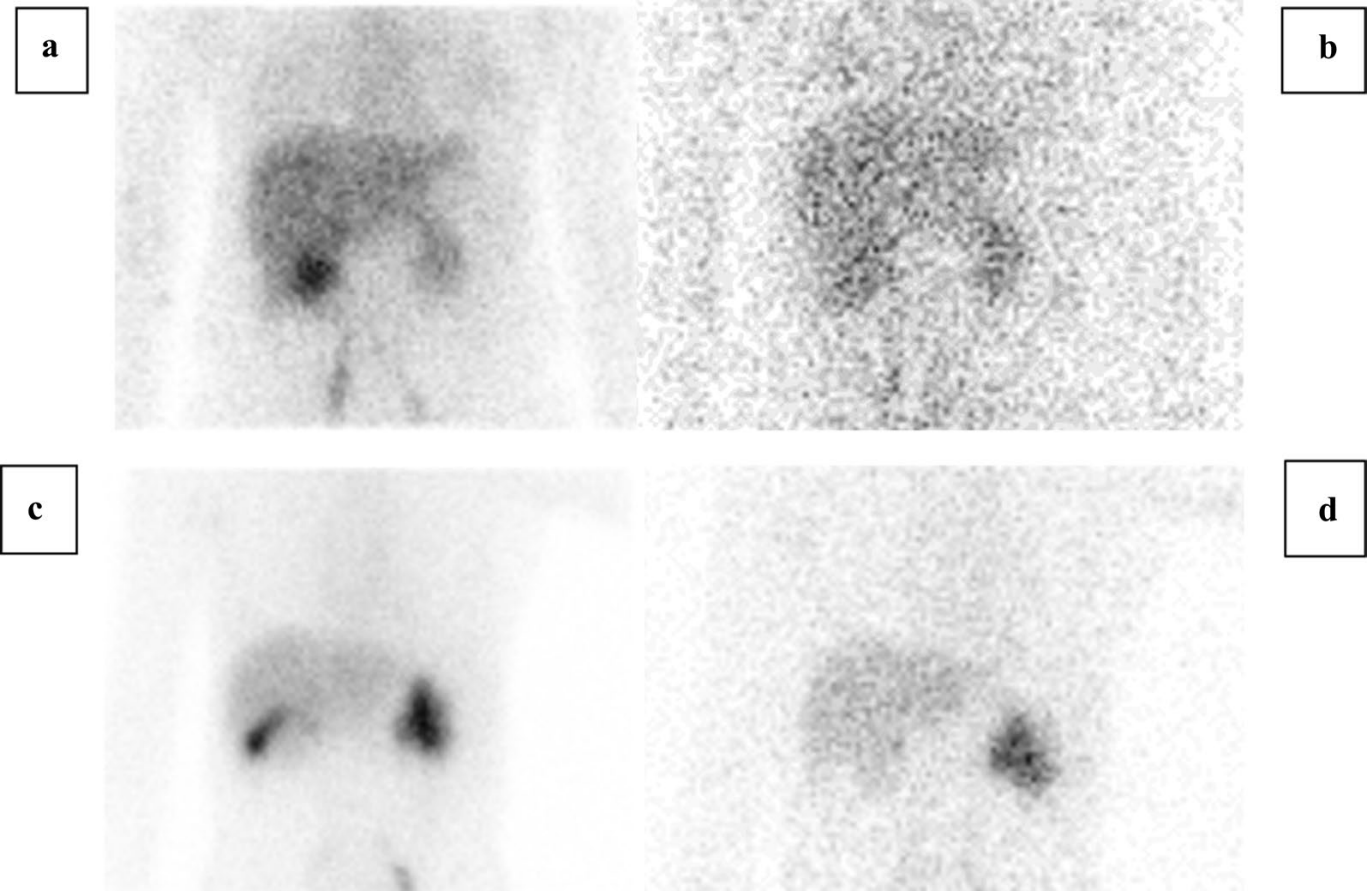
Anterior images obtained 30 min after injection demonstrate [^99m^Tc]Tc-MAG3 activity in the gallbladder of subjects 10 (**a**) and 13 (**c**) whereas the corresponding [^99m^Tc]Tc(CO)_3_(NTA) images (**b**) and (**d**) show no gallbladder activity

The serum creatinine was not available for two patients; the mean serum creatinine for the remaining 16 subjects was 1.17 ± 0.59 mg/dL with 4 subjects having reduced renal function (serum creatinine > 1.3 mg/dL) (Table 1). The clearance of NTA was significantly greater than the clearance of MAG3, 331 ± 146 versus 271 ± 105 mL/min/1.73 m^2^, respectively, *p* < 0.0001 (Table 1). In every kidney but one, the kidney to background ratio was greater for NTA than MAG3, mean of 6.2 versus 5.2 for the left kidney and 5.1 versus 4.0 for the right kidney, respectively, *p* < 0.001 (Table 2). Relative uptake was equivalent for the two tracers; the mean percent uptake (± SD) of NTA in the left kidney was 56.6 ± 17.6% compared to 56.4 ± 17.9% for MAG3, *p *= 0.75 (Table 1). Hydration was also comparable; there was no significant difference in the voiding volumes following the NTA and MAG3 acquisitions, 598 ± 237 mL versus 498 ± 170 mL, respectively, *p* = 0.07 (Table 1). In 2/18 patients, anterior images of the gall bladder 30 min after tracer injection failed to be obtained. In the remaining 16 patients, gallbladder activity was present in 6/16 MAG3 studies; no gallbladder activity was observed following NTA administration (Fig. 1).

**Table 1:** Dose injected (MBq) of [^99m^Tc]Tc(CO)_3_(NTA) and [^99m^Tc]Tc-MAG3, serum creatinine, voided volume, percent uptake and the camera based clearance

Subject	Dose injected (MBq)		Creatinine (mg/dL)	Voided volume (mL)		Left kidney (%)		Right kidney (%)		Clearance (mL/min/1.73 m^2^)	
	NTA	MAG3		NTA	MAG3	NTA	MAG3	NTA	MAG3	NTA	MAG3
1	44.8	345.2	0.49	*	*	52	53	48	47	526	368
2	312.3	44.4	0.98	360	350	80	82	20	18	123	72
3	46.2	337.8	0.5	1000	600	48	47	52	53	468	323
4	46.2	320.1	NA	800	850	NA	NA	100	100	380	276
5	234.2	46.2	1.08	600	600	25	26	75	74	252	252
6	39.6	321.9	0.74	850	700	79	75	21	25	438	378
7	48.1	345.2	0.9	*	*	52	49	48	51	396	306
8	51.1	312.3	1.93	320	400	NA	NA	100	100	85	85
9	54.4	315.2	0.7	*	*	49	47	51	53	341	295
10	52.2	358.9	1.9	*	*	88	90	12	10	226	191
11	47.7	321.5	1.27	*	*	77	79	23	21	340	313
13	54.4	319.7	0.79	800	380	50	50	50	50	513	389
14	55.1	302.3	NA	*	*	58	50	42	50	259	238
15	53.6	286.7	1.24	500	550	28	29	72	71	150	149
16	57.0	346.3	0.78	450	350	48	46	52	54	456	378
17	58.1	388.5	2.44	360	350	51	56	49	44	226	233
18	58.8	357.1	2.01	360	300	64	63	36	37	218	188
19	53.3	359.3	1.01	780	550	57	60	43	40	566	441
Mean	NC	NC	1.17	598	498	56.6	56.4	49.7	49.9	331	271
SD			0.59	237	170	17.6	17.9	24.7	24.8	146	105
*P*				0.07		0.75		0.75		0.0002	

NC, not calculated since two NTA and two MAG3 patients received high and low amounts of tracer activity, respectively

Patient 12 withdrew from the study prior to tracer injection

NA, not available

^*^Voiding volumes are not comparable since the patient voided prior to the completion of an acquisition

**Table 2:** Kidney to background ratios of NTA and MAG3

	Left kidney		Right kidney	
Subject	MAG3	NTA	MAG3	NTA
1	7.7	9.5	5.6	7.6
2	2.0	3.1	0.4	0.8
3	7.0	9.2	6.2	7.6
4	AK	AK	10.1	14.3
5	3.0	3.7	4.0	5.0
6	4.1	6.6	1.8	2.4
7	7.2	8.2	6.7	7.2
8	AK	AK	2.6	3.3
9	5.2	5.9	4.7	5.3
10	5.0	5.3	0.9	1.4
11	6.2	6.2	1.7	2.2
13	7.9	10.3	6.0	8.1
14	2.5	2.9	2.2	2.3
15	1.7	2.1	3.1	3.9
16	7.7	8.8	6.0	7.3
17	2.8	3.0	2.1	2.6
18	5.1	6.0	2.8	3.0
19	8.2	8.5	5.4	7.3
Mean	5.2	6.2	4.0	5.1
SD	2.3	2.7	2.5	3.4
*P*		0.0002		0.0003

Patient 12 withdrew from the study prior to tracer injection

AK, absent kidney

For the left kidney, the mean time to peak activity was less for NTA than MAG3, 2.6 ± 0.5 min versus 3.5 ± 1.4 min, respectively (*p *= 0.02). The mean time to peak activity did not attain significance for the right kidney, 4.0 ± 5.2 min versus 5.8 ± 5.6 min, respectively *p* = 0.07 (Table 3), but when the one kidney with time to peak activity greater than 20 min for both tracers was omitted, the mean time to peak for NTA was less than that of MAG3, 2.7 ± 0.5 min versus 4.8 ± 3.7 min, respectively, *p* = 0.045 (Table 3).

**Table 3:** Time to peak (min), 20 min/maximum and postvoid/maximum count ratios for the right and left kidneys following administration of NTA and MAG3

Subject	Left kidney TTP (min)		Right kidney TTP (min)		Left kidney 20 min/max		Right kidney 20 min/max		Left kidney PV/max		Right kidney PV/max	
	NTA	MAG3	NTA	MAG3	NTA	MAG3	NTA	MAG3	NTA	MAG3	NTA	MAG3
1	2.6	2.8	2.8	3.9	0.12	0.17	0.23	0.56	0.05	0.14	0.1	0.4
2	ID	ID	ID	ID	ID	ID	ID	ID	ID	ID	ID	ID
3	2.5	2.8	2.5	2.5	0.14	0.21	0.08	0.19	0.05	0.12	0.03	0.12
4	AK	AK	ID	ID	AK	AK	ID	ID	AK	AK	ID	ID
5	3.7	3.7	3.2	2.5	0.51	0.82	0.18	0.2	0.79	0.76	0.11	0.11
6	3.2	4	2.1	2.7	0.18	0.23	0.22	0.24	0.09	0.17	0.11	0.16
7	2	2.5	2.6	16	0.08	0.17	0.36	0.97	0.07	0.14	0.11	0.59
8	AK	AK	3.9	10.8	AK	AK	0.58	0.88	AK	AK	0.53	0.65
9	2.4	2.8	2.3	4.8	OT	OT	OT	OT	0.1	0.21	0.16	0.27
10	2.1	2.3	3.2	2.1	OT	OT	OT	OT	0.09	0.2	0.12	0.21
11	2.2	3.1	2.6	3	OT	OT	OT	OT	0.15	0.16	0.08	0.14
13	2.2	7.8	2.4	2.6	0.34	0.79	0.09	0.16	NA	NA	NA	NA
14	3.2	3.5	23.5	21.3	OT	OT	OT	OT	0.13	0.21	1.04	1.08
15	3	3.3	2.1	2.6	0.39	0.64	0.28	0.41	0.65	0.42	0.24	0.28
16	3.2	4.8	2.5	3.8	0.2	0.15	0.09	0.16	0.07	0.09	0.06	0.08
17	2.5	3.8	3.5	6.8	0.6	0.67	0.66	0.83	0.66	0.6	0.45	0.49
18	2.7	3.3	2.1	5.3	0.29	0.46	0.4	0.73	0.24	0.37	0.49	0.69
19	1.9	2.5	2.6	2.7	0.2	0.15	0.14	0.25	0.07	0.09	0.1	0.13
Mean	2.63	3.53	3.99	5.84	0.28	0.41	0.28	0.47	0.23	0.26	0.25	0.36
SD	0.53	1.35	5.23	5.55	0.17	0.28	0.19	0.31	0.26	0.20	0.27	0.29
*P*	0.0224		0.0677		0.0201		0.0031		0.2334		0.0046	

Patient 12 withdrew from the study prior to tracer injection

AK, absent kidney; ID, infiltrated dose; NA, the postvoid image was not acquired and the ratio could not be calculated; OT, patient had to void before the study was completed

**Fig. 2:**
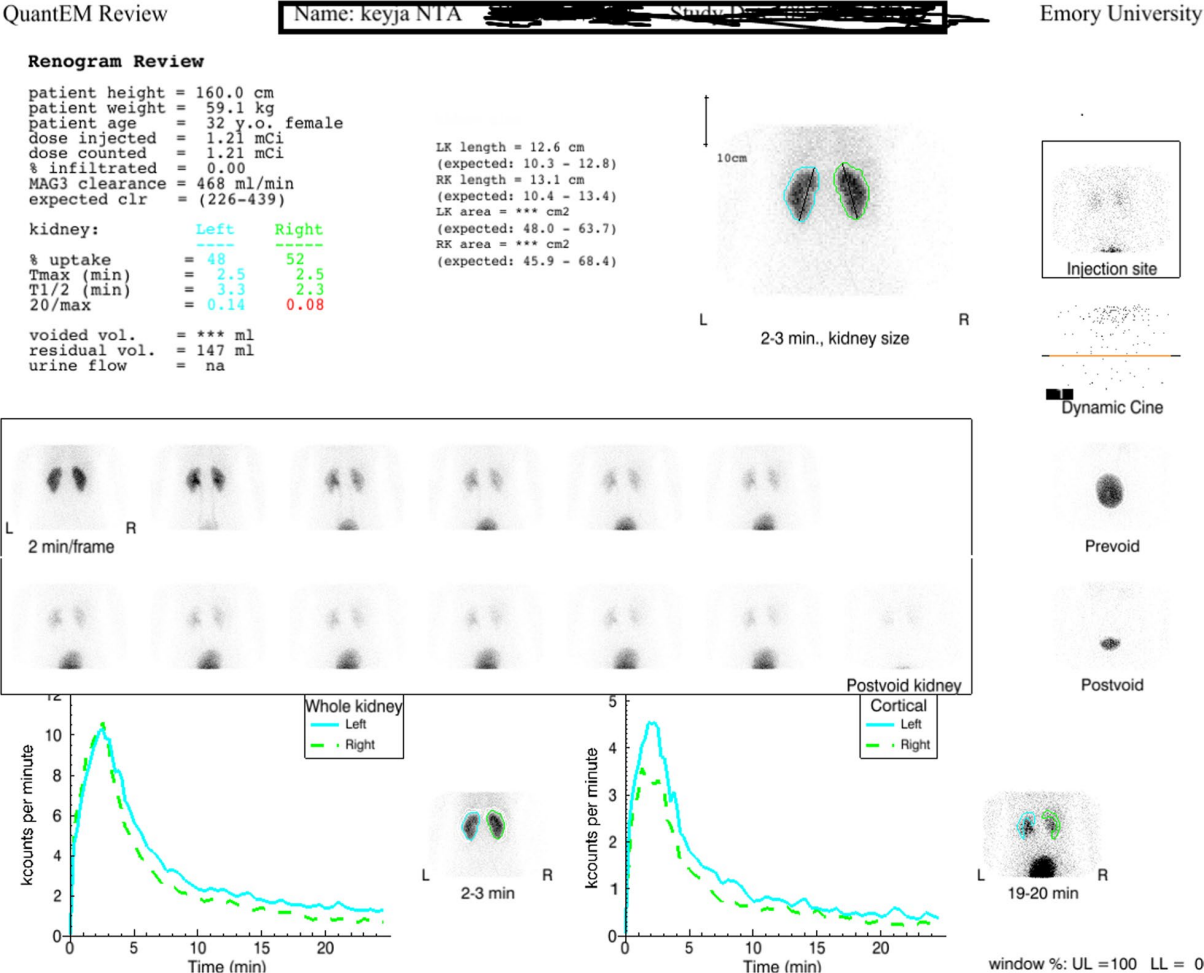
Subject 3: Two minute sequential images following the administration of [^99m^Tc]Tc-MAG3 (338 MBq) and [^99m^Tc]Tc(CO)_3_(NTA) (46 MBq)with the corresponding renogram curves. The T1/2 of the left kidney MAG3 was 5.5 min compared to 3.3 min for NTA

**Fig. 3:**
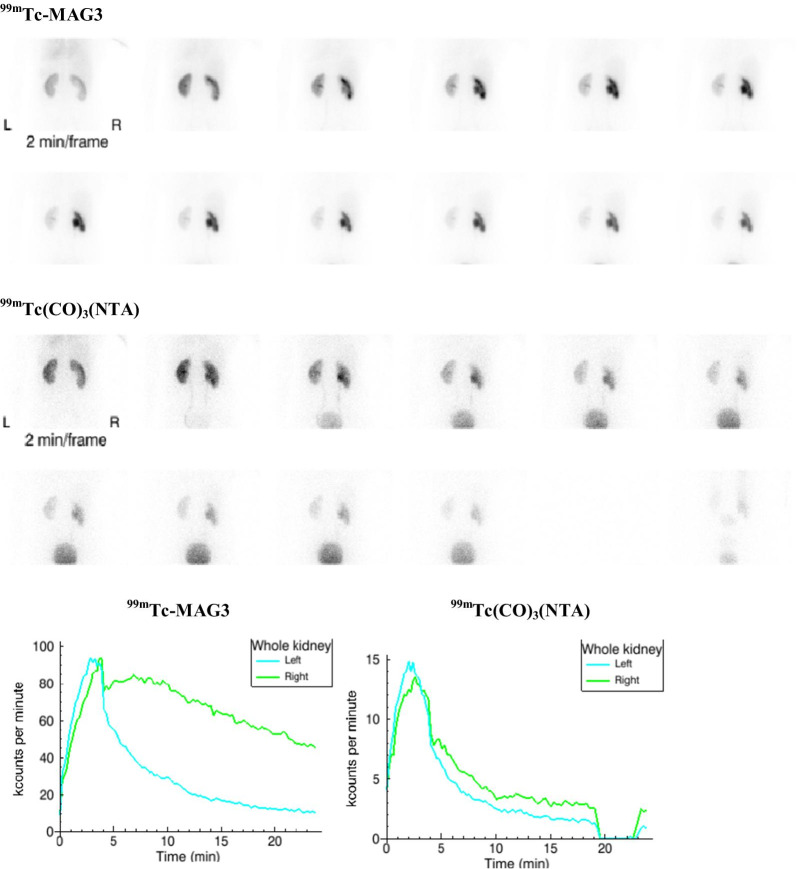
Subject 1: Two minute sequential images following the administration of [^99m^Tc]Tc-MAG3 (338 MBq) and [^99m^Tc]Tc(CO)_3_(NTA) (45 MBq) with the corresponding renogram curves. The subject receiving NTA had to get off the table to void at 20 min and was repositioned for the final frame. The relative uptake of the left kidney was 52% for NTA and 53% for MAG3. The T1/2 for ^99m^Tc-MAG3 was 17.6 min compared to 4.0 min for NTA. Because of the images, renogram curve and prolonged T1/2, the right kidney of the MAG3 acquisition might be interpreted as being indeterminate or representing an obstructed kidney whereas the NTA acquisition demonstrates that the right kidney is not obstructed

**Fig. 4:**
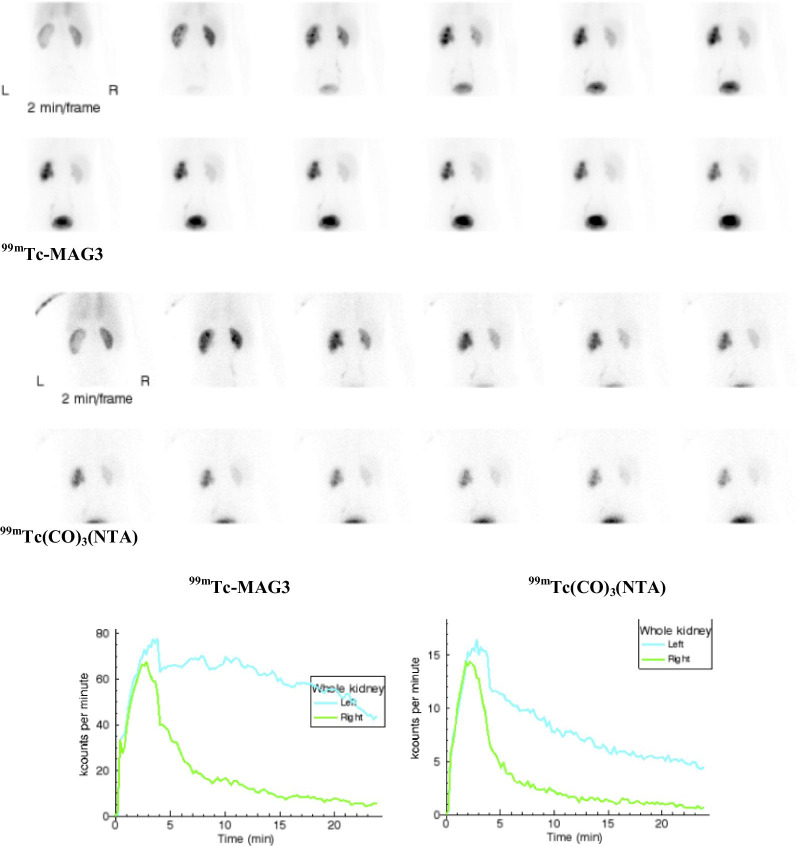
Subject 13: Two minute sequential images following the administration of [^99m^Tc]Tc-MAG3 (320 MBq) and [^99m^Tc]Tc(CO)_3_(NTA) (54 MBq) with the corresponding renogram curves. The relative uptake of the left kidney was 50% for both tracers. The T1/2 of the left kidney for MAG3 was > 50 min and the 20 min/maximum count ratio was 0.79 compared to a T1/2 of 7 min and 20 min/max count ratio of 0.34 for NTA. The images, renogram curve, prolonged T1/2 and elevated 20 min/max ratio of the MAG3 study might be interpreted as indeterminate for obstruction or representing an obstructed left kidney. In contrast, the combination of a normal T1/2, equal uptake by the two kidneys and a normal 20 min/max ratio for NTA study excludes left kidney obstruction

**Fig. 5:**
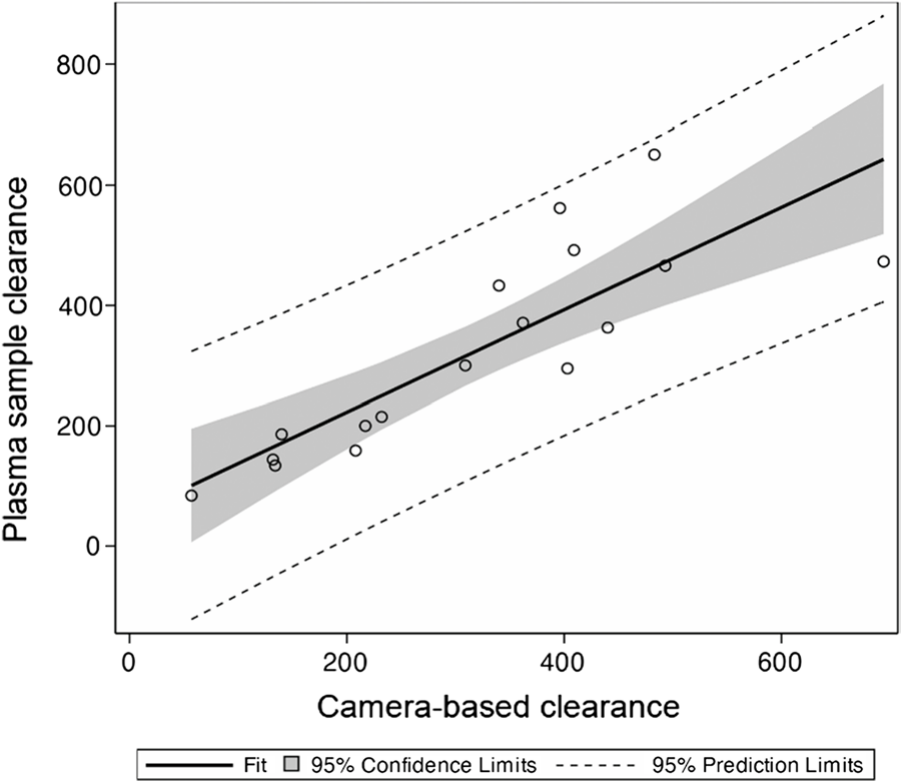
Plot comparing the camera-based and plasma sample clearances of NTA (mL/min/1.73 m^2^). The intercept is 52 (95% confidence intervals of − 50 to 154) and the slope is 0.85 (95% confidence intervals of 0.67–1.13)

The mean 20 min to maximum count ratios for NTA were lower than those of MAG3 for both the left and right kidneys, *p* = 0.02 and *p* = 0.003, respectively (Table 3). The mean postvoid/maximum count ratio, an important parameter for evaluating suspected obstruction, was significantly lower in the right kidney for NTA than MAG3, *p* = 0.005, although no difference was noted in the left kidney, *p* = 0.20 [22] (Table 3). Images and curve parameters for the two tracers were comparable for the majority of studies; an example is illustrated in Fig. 2. In two patients, MAG3 studies may have been interpreted as indeterminate or representing renal obstruction whereas the NTA studies excluded obstruction (Figs. 3, 4) [4, 22]. One-year follow up of these two patients confirmed the absence of obstruction; no procedure was performed and a repeat MAG3 study at one year showed no change in renal function. Finally, there was very good agreement between the camera-based and plasma sample NTA clearances, *r* = 0.837, with a 95% confidence interval 0.608–0.937 (Table 4, Fig. 5).

**Table 4:** Camera-based and plasma sample clearance of NTA (mL/min/1.73 m^2^)*

Subject	Camera-based clearance	Plasma sample clearance
1	140	186
2	232	215
3	403	295
4	208	159
5	217	200
6	57	84
7	132	144
8	134	134
9	440	363
10	309	300
11	694	473
12	493	466
13	340	433
14	396	562
15	483	651
16	362	371
17	409	492

^*^Data are derived from subjects in references [8] and [9]

## Discussion

Rapid drainage of tracer from the kidney before or after a diuresis stimulated by furosemide administration excludes obstruction but evaluation of drainage can become challenging when there is substantial uptake of circulating tracer during the washout period. Tracers with a high extraction efficiency and rapid clearance allow a more straightforward evaluation of drainage; they minimize the amount of circulating tracer entering the kidney during the washout phase of the renogram. It is particularly important to minimize contamination of the washout curve in patients with underlying kidney disease since these patients typically have a slower than normal transit of the tracer through the kidney. We chose to study patients with suspected renal obstruction because that group provides a clinically relevant population to for the comparison of NTA and MAG3.

Clearances were measured using a camera-based technique based on the cumulated renal activity 1–2.5 min after injection [18]. A higher clearance for NTA implies that the kidney has accumulated a higher percentage of the injected dose in the 1–2.5 min interval than the injected dose of MAG3. Optimally, a camera-based clearance regression equation for NTA would be based on a large population comparing percent dose in the kidney at 1–2.5 min post injection with a multisample plasma clearance as was done for MAG3; however, initial data from two earlier studies show that the regression equation developed forMAG3 does provide a good measurement of the NTA clearance.

NTA was given first in 16/18 studies; consequently, any NTA activity remaining in the kidney when MAG3 was administered would add to the renal activity measured at 1–2.5 min post injection and result in an overestimation of the MAG3 clearance. This error was probably small since NTA is rapidly eliminated and much more activity was administered for the MAG3 injection but it may have biased our results toward an overestimation of the MAG3 clearance. Despite this possibility, the NTA clearance was still significantly greater than that of MAG3. In fact, the clearance of NTA is equal to that of ^131^I-OIH and will provide an equivalent measure of effective renal plasma flow [8, 9].

Background correction is of crucial importance for camera-based clearance methods since background counts may be relatively high during the first minutes of the study when camera-based clearances are calculated. For example, background counts during the 2nd–3rd minutes following DTPA administration are reported to reach 50–80% of the total non-corrected renal activity [23]. High background counts increase the difficulty of an accurate background correction and may compromise the accuracy of a camera-based clearance. NTA has a higher extraction efficiency than either DTPA or MAG3 and a higher kidney to background ratio; consequently, use of NTA will minimize the error associated with background correction, allow a more accurate assessment of renal activity and will likely result in more robust camera-based clearance measurements with better reproducibility. The lack of hepatobiliary elimination of NTA should also contribute to the accuracy and reproducibility of camera-based clearance methods.

The majority of patients received 40 mg of furosemide 15 min prior to the initial tracer injection. Six of 18 patients (33%) had to void prior to completion of the 24-min acquisition. These results are similar to those reported by Liu et al. where 30% of patients also failed to complete a 30-min acquisition following an F-15 protocol [24]. In normal subjects, 40 mg of furosemide produces a maximal diuresis; the onset of diuresis begins almost immediately and reaches 80% of the maximum within 3–6 min of injection [17, 25]. Use of the F-15 min protocol with the standard doses of furosemide increases the likelihood that bladder capacity will be overloaded and the patient will have to void before completing the acquisition.

The images and renogram curves were comparable for the majority of the studies. Depending on the criteria used, the MAG3 study may have been interpreted in two patients as showing one kidney to be indeterminate for obstruction or possibly obstructed. In contrast, the NTA acquisition in these patients clearly excluded obstruction. The absence of obstruction was confirmed on follow up. Neither patient had an intervention and repeat diuretic renography studies one year later showed stable renal function.

There are several limitations. Our intention was to alternate the order of NTA and MAG3 administration but, as explained earlier, NTA was administered first in 16/18 studies and residual activity in the kidney may have led to an overestimation of the MAG3 clearance. Our protocol was designed to equalize the plasma concentrations of furosemide prior to each tracer administration in an attempt to equalize the diuretic responses; however, 33% of patients had to void prior to completion of the first acquisition whereas only 11% failed to complete the second acquisition. These results suggest that the diuretic response may have been greater following the NTA administration although there was an excellent diuretic response following each tracer and there was no significant difference in the voided volumes of patients who completed both acquisitions. Finally, future efforts need to focus on developing a kit formulation for NTA to provide broader availability.

## Conclusion

NTA has a more rapid clearance than MAG3 and results in higher kidney to background ratios. The higher kidney to background ratios of NTA will minimize error associated with background subtraction and should result in more robust and reproducible camera-based clearance measurements. Unlike MAG3, there was no elimination via the hepatobiliary tract. Moreover, NTA may be able to exclude obstruction in selected patients when MAG3 studies are indeterminate or may even suggest an obstructed kidney. Finally, use of the F-15 min protocol with the standard 40 mg doses of furosemide increases the likelihood that the patient will need to void prior to completing the acquisition.

## Data Availability

Relevant data can be provided pending a justified request.
